# Recent advances in regulatory immune cells: exploring the world beyond Tregs

**DOI:** 10.3389/fimmu.2025.1530301

**Published:** 2025-05-16

**Authors:** Peng Shi, Yi Yu, Hongwei Xie, Xiaofang Yin, Xue Chen, Yongfei Zhao, Hai Zhao

**Affiliations:** ^1^ Department of Emergency, the Affiliated Hospital of Qingdao University, Qingdao, Shandong, China; ^2^ Department of Neurosurgery, the Affiliated Hospital of Qingdao University, Qingdao, Shandong, China

**Keywords:** Tregs, CD8+ Tregs, Bregs, MDSC, DCregs, ILCregs, NKregs

## Abstract

Regulatory immune cells are pivotal in maintaining immune homeostasis and modulating immune responses to prevent pathologies. While T regulatory cells (Tregs) are extensively recognized for their immunosuppressive roles, emerging subsets of regulatory cells, including regulatory CD8+ cells (CD8+Tregs) regulatory B cells (Bregs), myeloid-derived suppressor cells (MDSCs), regulatory dendritic cells (DCregs), regulatory innate lymphoid cells (ILCregs), and regulatory natural killer cells (NKregs), are garnering increased attention. This review delves into the phenotypic characteristics, mechanisms of action, and immune-regulatory functions of these lesser-known but crucial immune cell subsets. The review provides a comprehensive examination of each cell type, detailing their origins, unique functionalities, and contributions to immune homeostasis. It emphasizes the complex interplay among these cells and how their coordinated regulatory activities influence immune responses in diverse pathological and therapeutic contexts, including autoimmunity, cancer immunotherapy, chronic inflammation, and transplant tolerance. By unraveling these mechanisms, the review outlines novel therapeutic avenues, such as targeting these regulatory cells to modulate immune activity and enhance precision medicine approaches. The future of immunotherapy and immune modulation lies in leveraging the expanded knowledge of these regulatory immune cells, presenting challenges and opportunities in clinical applications.

## Introduction

Recent advancements in immunology have underscored the pivotal role of regulatory immune cells in orchestrating immune responses and maintaining immune homeostasis (1–5). These specialized subsets of immune cells possess unique immunoregulatory functions, modulating the activity of various immune effectors to prevent autoimmunity, limit inflammation, and facilitate tissue repair (2, 6–9). Understanding the intricate interplay between regulatory immune cells and the broader immune system is paramount for deciphering the pathogenesis of immune-related disorders and devising novel therapeutic strategies.

T regulatory cells (Tregs) represent a cornerstone in the realm of regulatory immune cells. Initially identified for their role in immune tolerance and prevention of autoimmunity, Tregs have garnered substantial attention due to their diverse functional repertoire and plasticity (10–12). Historic discoveries elucidating the crucial function of Tregs in maintaining immune balance have been complemented by recent insights into their heterogeneity, tissue-specific localization, and crosstalk with other immune cell subsets. The evolving landscape of Tregs biology continues to unravel novel mechanisms underlying immune regulation and their implications in health and disease (13).

Beyond Tregs, a myriad of other regulatory immune cell populations has emerged as key players in immune modulation. CD8+ Tregs are a specialized subset of T lymphocytes expressing the CD8 co-receptor and they suppress immune responses through mechanisms such as targeted cytotoxicity against activated immune cells, secretion of anti-inflammatory cytokines (e.g., IL-10, TGF-β), and direct inhibition of effector T cells, uniquely regulating CD8+ T cell-driven immunity while maintaining peripheral tolerance (14). Regulatory B cells, characterized by their ability to produce anti-inflammatory cytokines and induce T cell tolerance, represent a burgeoning field of study with implications in autoimmune diseases and cancer immunotherapy (15). The Treg-of-B cells are a unique subset of regulatory T cells generated by B cells, notable for their lack of FOXP3 (Forkhead box P3) expression, setting them apart from conventional regulatory T cells (16). These cells are characterized by markers such as LAG3 (Lymphocyte-activation gene 3), ICOS (Inducible T-cell co-stimulator), PD1 (Programmed cell death protein 1), GITR(Glucocorticoid-induced TNFR-related protein), and CTLA4 (Cytotoxic T-lymphocyte-associated protein 4) and primarily exert their regulatory function through cell-cell contact mechanisms, rather than cytokines like IL-10 (Interleukin-10). While they do produce IL-10, it is not essential for their suppressive activity, which operates through both IL-10-dependent and independent pathways. This unique profile suggests that Treg-of-B cells contribute to immune tolerance through mechanisms distinct from conventional Tregs (17, 18). MDSCs, DCregs, ILCregs, and NKregs collectively contribute to the intricate network of immune regulation, each exerting unique suppressive functions and immune-modulating properties in diverse pathological contexts (19–23).

As research into regulatory immune cells advances, several challenges and opportunities lie ahead. Unraveling the complexities of regulatory cell subsets, deciphering their precise mechanisms of action, and elucidating their crosstalk within the immune microenvironment pose formidable tasks. Moreover, translating fundamental insights into clinical applications necessitates overcoming hurdles related to cell-based therapies, biomarker identification, and patient stratification. Nonetheless, the burgeoning field of regulatory immune cells holds promise for revolutionizing immunotherapy and ushering in a new era of precision medicine in immune-mediated disorders.

## Advancements in understanding regulatory immune cells

In recent years, significant strides have been made in elucidating the intricate roles of regulatory immune cells in modulating immune responses and maintaining immune homeostasis. These advancements have revolutionized our understanding of the immune system and its regulatory mechanisms, shedding light on diverse cell populations beyond Tregs. Research efforts have uncovered a myriad of regulatory immune cell subsets, each endowed with distinct functions and regulatory capacities. From the discovery of regulatory B cells to the emerging insights into the regulatory potential of innate lymphoid cells, our comprehension of these cells continues to evolve rapidly (24). We will provide an overview of the recent advancements in understanding regulatory immune cells, highlighting their diverse functions, regulatory mechanisms, and implications for immune-related diseases and therapeutic interventions. In exploring the landscape of regulatory immune cells, one cannot ignore the significant contributions and insights derived from Treg research, which continue to shape our understanding of immune regulation.

### Regulatory T cells

Although Tregs are not the central theme here, their pivotal role in the broader context of regulatory cells cannot be overlooked in any comprehensive research of immune regulation.

**Figure 1:**
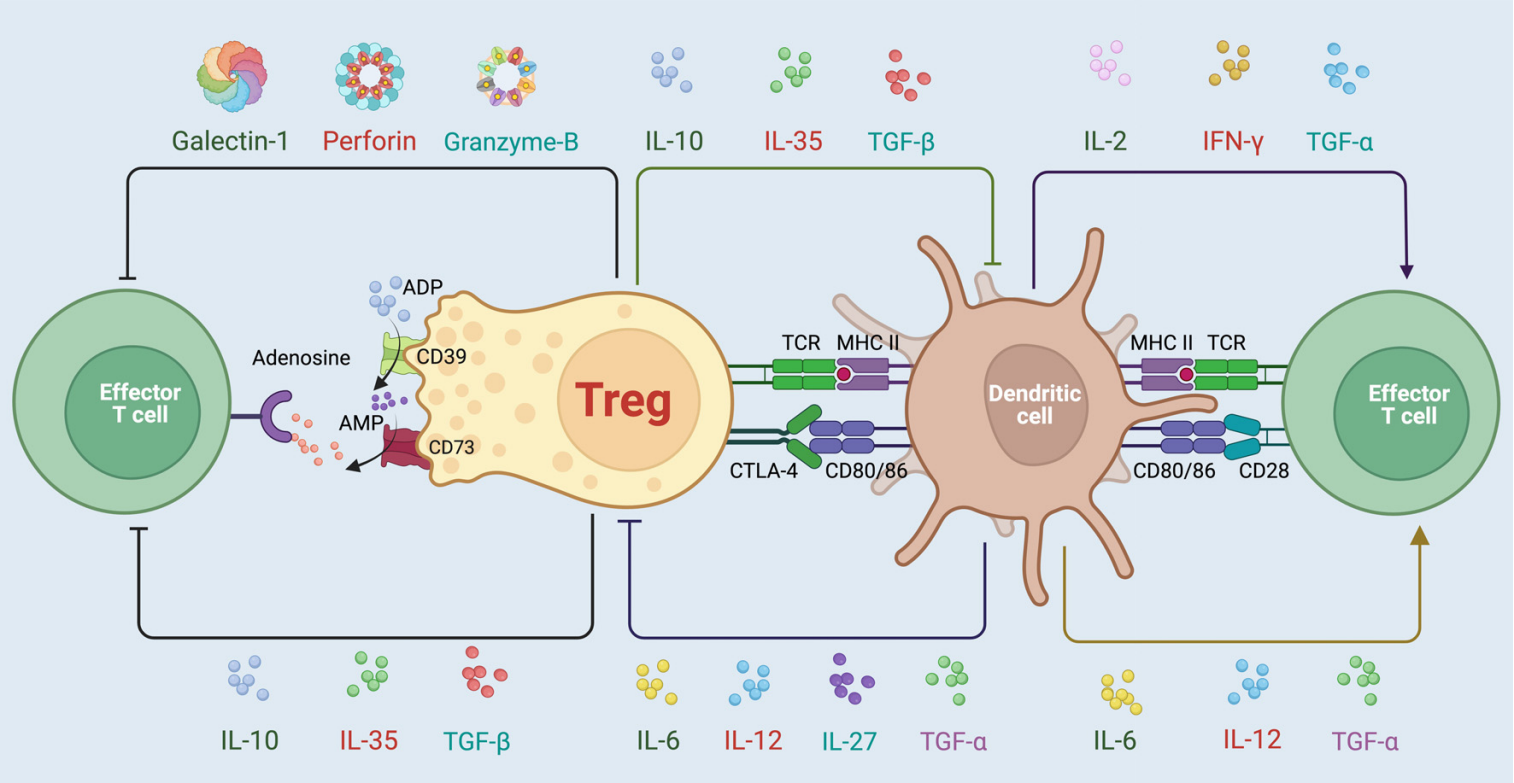
Variable mechanisms involved in the immunosuppressive activities mediated by Tregs. Tregs employ a diverse array of strategies to maintain immune homeostasis and suppress overactive immune responses. One primary mechanism involves the secretion of immunosuppressive cytokines such as IL-10, TGF-β, and IL-35, which directly inhibit the activation and proliferation of effector T cells (13). Additionally, Tregs modulate immune responses through cell surface interactions, notably via the expression of CTLA-4, which competes with CD28 for CD80/86 on dendritic cells, dampening their ability to activate effector T cells (26). Tregs also exert influence through metabolic disruption, utilizing CD39 and CD73 to convert extracellular ATP to adenosine, further suppressing effector cell function via adenosine receptor signaling (27, 28). Furthermore, Tregs can release cytotoxic molecules like Galectin-1, Perforin, and granzyme B, contributing to the direct elimination of pathologically active cells (29, 30). Through these complex and coordinated actions, Tregs play a critical role in preventing autoimmune diseases, controlling chronic inflammation, and promoting tolerance to self-antigens and transplanted tissues.

Conventional Tregs have long been recognized for their pivotal role in maintaining immune homeostasis by suppressing inappropriate immune responses (25). This capacity to modulate immune responses is critical not only in preventing autoimmune diseases but also in controlling inflammation and promoting tolerance across various biological systems (13) (**Figure 1**). Recent studies have expanded our understanding of Tregs functions beyond their traditional roles (31). These cells are now known to engage in several non-immune functions that are crucial for maintaining tissue homeostasis. For instance, Tregs have been implicated in metabolic regulation, particularly in adipose tissues where they influence insulin sensitivity and lipid metabolism (32). Tregs are alos influenced by lipid metabolism, particularly through the PPAR-γ (Peroxisome proliferator-activated receptor-γ) receptor, which is sensitive to lipid interactions that can impair Tregs functionality (33). Moreover, Tregs contribute to the maintenance of stem cell niches, such as those found in the bone marrow, skin, and intestines (34, 35). By modulating the local microenvironment, Tregs can protect stem cells from oxidative stress and promote their quiescence, which is crucial for long-term tissue regeneration (36). This interaction also highlights the broader role of Tregs in tissue repair and regeneration, where they can directly affect tissue cells to promote healing and restoration after injury. Furthermore, Tregs have been shown to facilitate tissue repair by producing growth factors and cytokines that directly interact with tissue cells. These molecules help in the proliferation and function of cells involved in tissue repair, such as epithelial cells in the skin and lung, and satellite cells in muscle tissue (2, 37).

In the context of clinical applications, the expanded understanding of Treg functions opens new avenues for therapeutic interventions aimed at modulating Treg activity. By targeting the non-canonical functions of Tregs, it is possible to develop treatments that enhance their regulatory capabilities, thereby improving outcomes in diseases characterized by inflammation, autoimmunity, or impaired tissue repair. These insights into the multifaceted roles of Tregs underscore their importance not only in immune regulation but also in broader physiological processes, making them a key target for future research and therapeutic development (1, 38).

When discussing regulatory T cells, it is increasingly imperative not to overlook the presence and significance of CD8+ Tregs. These cells represent a unique subset of T cells with critical implications in maintaining immunological tolerance and modulating the immune environment in various diseases, including cancer (39–41). They exhibit a capacity to suppress immune responses, which is pivotal in preventing autoimmunity but can also facilitate tumor immune evasion by weakening antitumor immune attacks (42). Research has elucidated distinct properties and mechanisms of CD8+ Tregs that differentiate them from their CD4+ counterparts (43). Predominantly, CD8+ Tregs exert their regulatory functions through the expression of molecules like Foxp3, CTLA-4, and others which are shared with CD4+ Tregs, yet they also display unique markers such as CD122 and CD28⁻, reflecting their distinct regulatory pathways (44). These cells are found elevated in various cancers, where they contribute to an immunosuppressive microenvironment that promotes tumor growth and survival by inhibiting effective immune surveillance and clearance. Interestingly, these cells not only modulate immune responses through direct cell-cell interactions but also through the secretion of immunosuppressive cytokines and metabolic disruption of effector cells (45–48). The induction of these Tregs can be driven by the tumor-derived factors and by the tumor microenvironment itself, which alters T cell metabolism and differentiation paths, skewing them towards a regulatory phenotype. The profound impact of CD8+ Tregs in cancer suggests that they could serve as a potential target for therapeutic intervention (49). Modulating the function or abundance of these cells could enhance the efficacy of cancer immunotherapies, providing new avenues to improve patient outcomes in oncological treatments. Understanding and manipulating the balance of Treg activity in the tumor context is therefore crucial for developing more effective cancer immunotherapies (50).

### Regulatory B cells

Bregs (Regulatory B cells) are a specialized subset of B lymphocytes that play a crucial role in immune regulation and maintaining immune homeostasis. They are known for their ability to suppress immune responses and promote immune tolerance. Researchers have made significant progress in identifying and characterizing Bregs. They have identified specific cell surface markers and functional properties that distinguish Bregs from other B cell subsets, which helps in understanding their unique regulatory functions (51).

Establishing the existence of one or multiple subsets of Bregs has been challenging due to the absence of specific markers that are analogous to the Treg marker FoxP3 (51, 52). The presence of multiple reported phenotypes for Bregs suggests the potential existence of distinct subsets. However, there are common surface markers shared among the proposed Breg subsets, which suggests the possibility that all Bregs originate from a common precursor (53, 54). The observed differences in phenotype could be attributed to the activation of this common Breg precursor in different microenvironments.

Specific markers for Bregs include (1). CD19: It is important to note that CD19 expression alone is not specific to Bregs, as it is also present on other B cell subsets. It is typically used in combination with other markers to define and isolate regulatory B cell subsets more precisely (51). (2). CD1d: CD1d, a molecule involved in lipid antigen presentation, plays a significant role in the context of Bregs. CD1d allows Bregs to directly interact with NKT cells through the presentation of lipid antigens via CD1d. Then Bregs can influence the function and activity of NKT cells (55). (3). CD5: In some studies, CD5 expression on B cells is commonly associated with B1 cells, a subset of B cells involved in innate-like immune responses and regulatory functions (56). CD5^+^ Bregs can produce regulatory cytokines such as IL-10 and could suppress immune responses and promote immune tolerance. While CD5^+^ Bregs have been well-characterized in mice, their presence and significance in human Bregs are still under investigation (57). (4). CD24: High expression of CD24 is often used as a defining feature and a marker for identifying Bregs in various contexts. CD24^+^ Bregs have been shown to possess potent immunosuppressive properties, including the production of regulatory cytokines such as IL-10 and the ability to suppress excessive immune responses. Modulating the function or increasing the numbers of CD24+ Bregs could be explored as a strategy for treating immune-related disorders, including autoimmune diseases and transplantation rejection (58). (5). CD38: Bregs with regulatory properties have been found to exhibit higher levels of CD38 compared to other B cell subsets. This suggests that CD38 can be used as a phenotypic marker to identify Bregs with immunosuppressive potential. CD38-expressing Bregs have been implicated in various disease contexts (59–61). For example, in autoimmune diseases like RA and SLE (systemic lupus erythematosus), decreased numbers or impaired function of CD38+ Bregs have been observed (62, 63). This suggests that CD38-expressing Bregs may play a role in maintaining immune tolerance and preventing excessive inflammatory responses (58). (6). CD27: The relationship between CD27 expression and Bregs is complex and can vary depending on the context and species studied. These CD27+ Bregs are believed to possess regulatory functions and contribute to immune tolerance. CD27 expression on Bregs has been associated with enhanced suppressive activity and the production of anti-inflammatory cytokines such as IL-10 and TGF-β. Some memory B cells with regulatory functions have been identified within the CD27+ B cell subset. These memory Bregs have been implicated in the regulation of immune responses and the maintenance of immune homeostasis (52).

**Figure 2:**
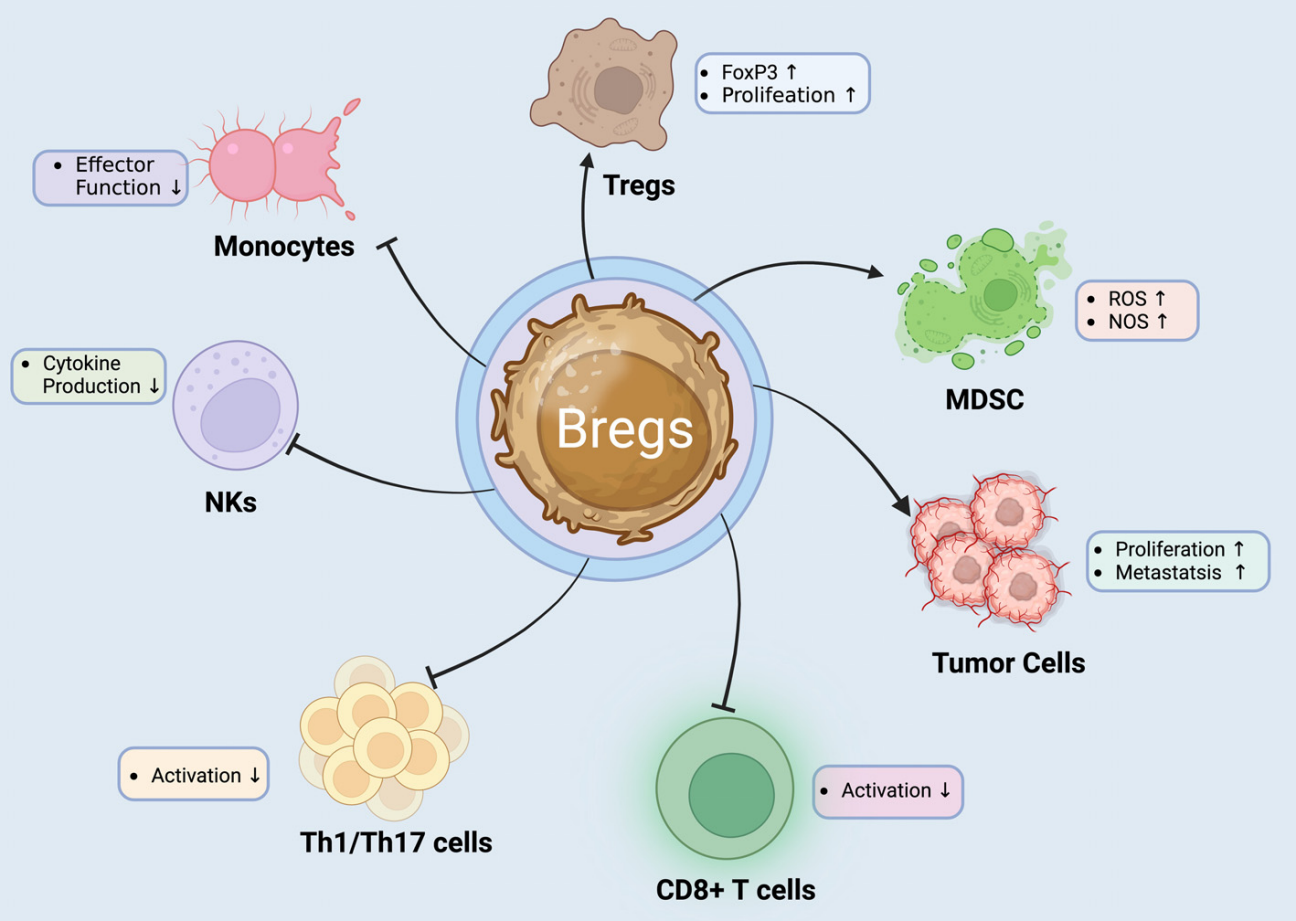
Regulatory B Cells: Orchestrators of Immune Suppression and Modulation. Bregs play a critical role in immune regulation by exerting suppressive effects on various immune cell types. They promote the proliferation and FoxP3 expression of Tregs, enhancing their immunosuppressive functions. Bregs also impact the tumor microenvironment by increasing ROS and NOS levels in MDSCs, which can aid tumor progression (55, 64). Additionally, Bregs inhibit the effector functions of NK cells and monocytes, reducing cytokine production and thus dampening inflammatory responses. Furthermore, Bregs decrease the activation of CD8+ T cells and Th1/Th17 cells, which are crucial for mediating autoimmune and inflammatory reactions. This multifunctional regulatory capacity makes Bregs a target of interest in developing therapies for autoimmune diseases and cancer immunotherapy (55).

Bregs employ various mechanisms to suppress immune responses and promote immune tolerance (**Figure 2**). Initially, Bregs are known to produce immunosuppressive cytokines, such as IL-10 and TGF-β. These cytokines play a crucial role in dampening immune responses by inhibiting the activation and function of effector immune cells. IL-10, in particular, is a potent anti-inflammatory cytokine that can inhibit pro-inflammatory cytokine production and immune cell activation (65, 66). Furthermore, Bregs can act as APCs and present antigens to T cells. However, instead of inducing T cell activation, Bregs often promote tolerance by inducing T cell anergy or Treg differentiation (67, 68). Moreover, Bregs have been shown to interact with and promote the generation and function of Tregs which are key mediators of immune tolerance and can suppress immune responses. Bregs can induce the differentiation and expansion of Tregs through cell-cell contact and the production of regulatory cytokines like IL-10 and TGF-β (65, 69, 70). Additionally, Bregs can exert suppressive effects through direct contact with immune cells. For example, Bregs can engage with and inhibit the activation of APCs such as DCs, thereby reducing their ability to stimulate immune responses. Bregs also directly interact with and suppress the function of effector T cells (71). Finally, Bregs can regulate immune responses by modulating the expression of co-stimulatory molecules. They can downregulate the expression of co-stimulatory molecules on APCs, which is essential for efficient T cell activation. By dampening the co-stimulatory signals, Bregs contribute to the suppression of immune responses (72, 73). Apart from IL-10 and TGF-β, Bregs can secrete other anti-inflammatory factors such as IL-35 and granulocyte-macrophage colony-stimulating factor (GM-CSF). These factors can suppress the activity of immune cells and promote immunosuppression (74, 75).

### Myeloid-derived suppressor cells

MDSCs are a heterogeneous population of immature myeloid cells with potent immunosuppressive capabilities. They play a crucial role in regulating immune responses in various pathological conditions, including cancer, infections, autoimmune diseases, and chronic inflammation (76–81). MDSCs are characterized by their myeloid origin, early differentiation stage, and ability to suppress immune responses through multiple mechanisms.

MDSCs arise from myeloid progenitor cells in the bone marrow. Under certain pathological conditions, such as cancer or inflammation, the differentiation of these cells is disrupted, leading to the accumulation of immature myeloid cells with suppressive functions in peripheral tissues (82, 83). MDSCs lack mature markers of myeloid cells and often express a combination of markers associated with early myeloid progenitors, including CD11b and Gr-1 in mice (84, 85). In humans, MDSCs are commonly identified by the expression of CD11b and CD33, as well as the lack of markers associated with mature immune cells, such as HLA-DR (86, 87). However, it is essential to note that MDSC phenotypes can vary depending on the context and the specific tissue involved.

**Figure 3:**
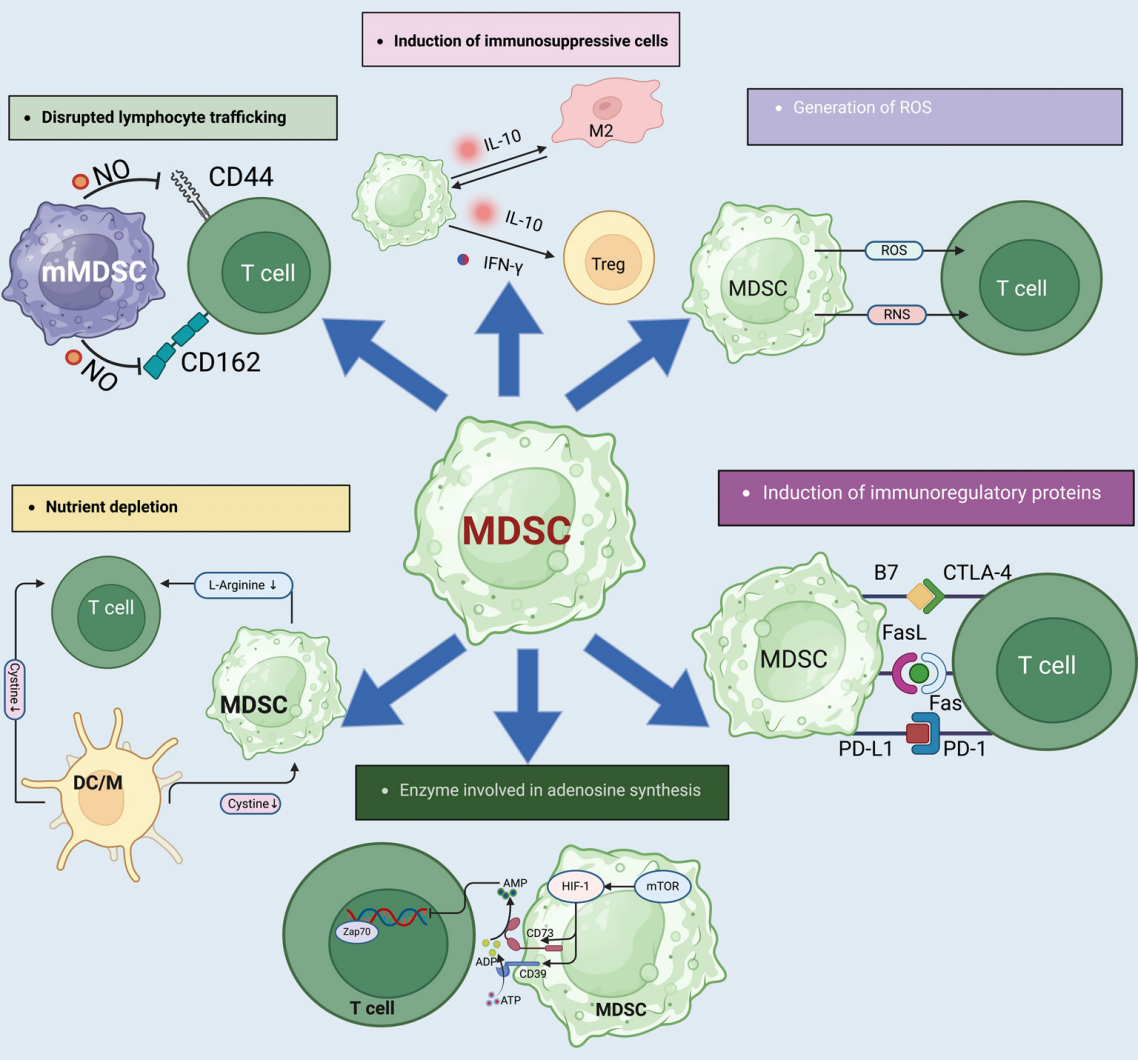
The complex mechanisms by which MDSCs influence immune suppression. (1). MDSCs also contribute to immune suppression by the impairment of T-cell homing to lymphoid tissues via interactions with selectins and cellular adhesion molecules like CD44 and CD62L. This impairment is facilitated by NO which alters T-cell migration, impacting immune surveillance and response. (2). MDSCs express ARG1 which depletes L-arginine, a critical molecule for T-cell receptor (TCR) expression and T-cell function. MDSCs can lead to reduced protein synthesis and glutathione production in T-cells, weakening the immune response. (4). Adenosine production is regulated by CD39 and CD73 ectoenzymes on MDSCs, which convert ATP to adenosine under hypoxic conditions. This adenosine then inhibits T-cell activation via suppression of kinase pathways, further contributing to the immunosuppressive microenvironment. (5). MDSCs contribute to immune suppression by producing IL-10 and IFN-γ. In addition, MDSCs downregulate pro-inflammatory cytokines like IL-6 and TNF-α in M2 macrophages, reinforcing a suppressive environment. (6). Free radicals, particularly reactive oxygen species (ROS) and reactive nitrogen species (RNS), are produced by MDSCs through enzymes like arginase-1 (ARG1), NOX2, and NOS2. These radicals inhibit T-cell function by inducing T-cell energy loss and promoting apoptosis, further contributing to immunosuppression. (7). MDSCs and Tregs express and activate inhibitory molecules like PD-L1, CTLA-4, and B7, which interact with PD-1 and CD28 on T cells to inhibit their activation and induce apoptosis.

MDSCs suppress immune responses through various mechanisms, contributing to immune evasion in cancer and other diseases. To begin with, MDSCs can secrete factors like IL-10, IL-6, IL-1β, TGF-β, and arginase-1, which dramatically increase the rate of accumulation and T cell suppressive activity of MDSC (88–92). Furthermore, MDSCs can trigger apoptosis in activated T cells through the production of reactive oxygen species (ROS) and other pro-apoptotic factors (93–95). Additionally, MDSCs consume and deplete essential nutrients such as arginine, tryptophan and cysteine, restricting their availability for T cell function (96–99). Moreover, MDSCs can impair the function of dendritic cells and macrophages, leading to decreased antigen presentation and diminished T cell activation (100–102) (**Figure 3**).

MDSCs can be further classified into two main subsets based on their phenotype in mice: Granulocytic MDSCs (G-MDSCs) express high levels of Ly6G and Ly6C and are morphologically similar to neutrophils (103, 104). Monocytic MDSCs (M-MDSCs) cells are characterized by high expression of Ly6C and low expression of Ly6G in mice, resembling monocytes (105, 106). In humans, the classification of MDSC subsets is more complex and remains an area of ongoing research.

MDSCs play a critical role in promoting tumor progression and immune evasion in cancer. Their accumulation is associated with poor prognosis in cancer patients (107–110). Moreover, MDSCs have been implicated in the pathogenesis of various inflammatory and autoimmune diseases, contributing to the dysregulation of immune responses (111–119).

### Regulatory DC cells

DCs (dendritic cells) are specialized antigen-presenting cells derived from the bone marrow that play a crucial role in initiating and regulating innate and adaptive immune responses. Regulatory or “tolerogenic” DCs are particularly important especially in the context of maintaining self-tolerance in a healthy state. These Dcregs employ various mechanisms to suppress or redirect the responses of naïve or memory T cells. In animal models of autoimmune diseases and transplant rejection, DCregs have demonstrated the ability to induce or restore T cell tolerance (120–124). Moreover, there is compelling evidence indicating that the transfer of DCregs can effectively modulate T cell responses not only in non-human primates but also in human subjects (120, 125). Building upon insights gained from *in vitro* experiments and animal models, efforts have been made to develop clinical-grade DCregs for the treatment of autoimmune diseases (120, 126–128). Clinical trials in Phase I evaluating the use of regulatory dendritic cell therapy in type-1 diabetes, rheumatoid arthritis and Crohn’s disease have shown promising results, demonstrating the feasibility and safety of this approach (129–132).

**Figure 4:**
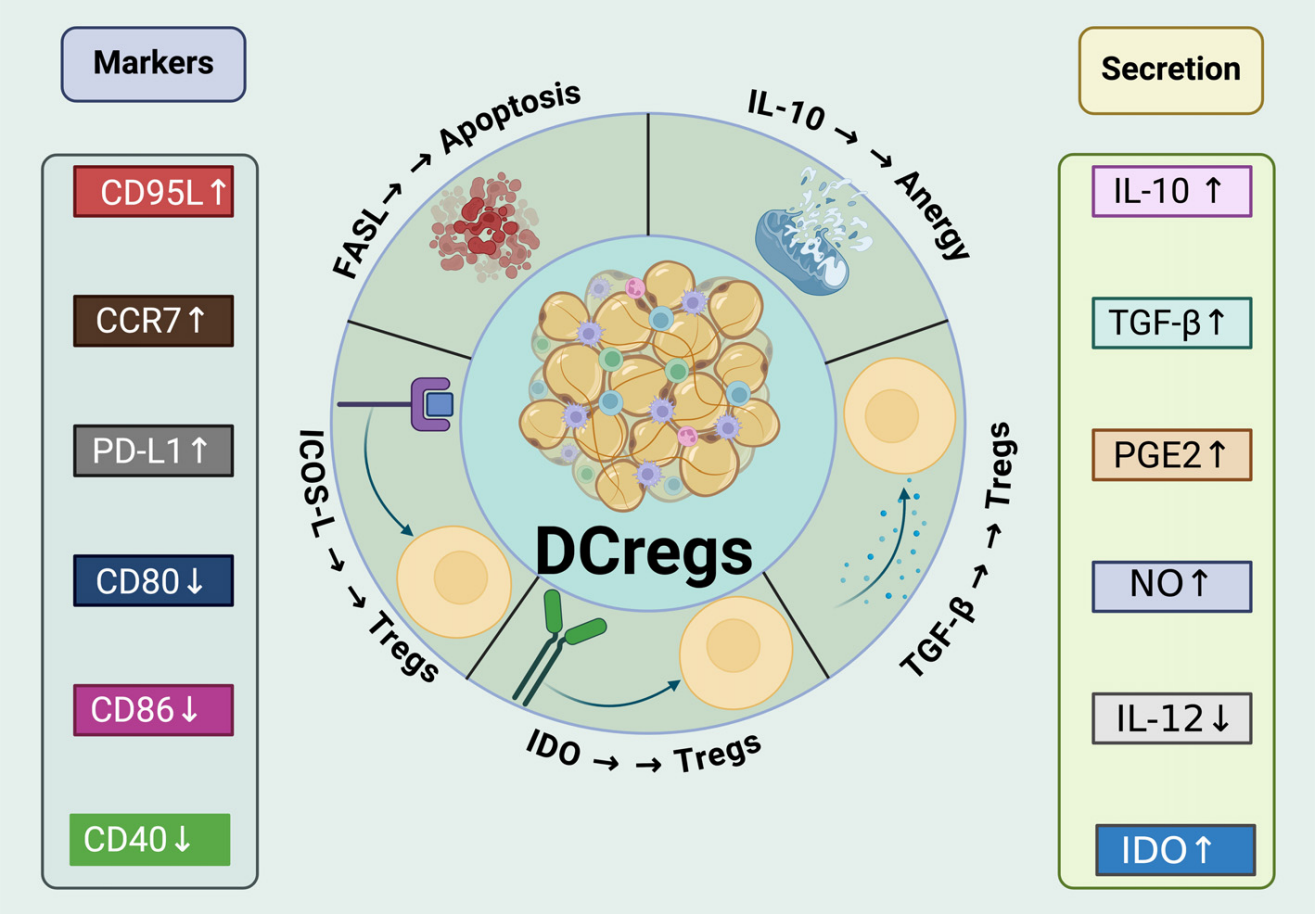
Mechanisms of immunosuppressive and tolerogenic activities of DCregs. DCregs express a variety of surface markers that are critical for their interaction with T cells and other immune cells. Markers like CD95L, CCR7, PD-L1, CD80, and CD86 are differentially expressed to modulate the immune response. The increased expression of CD95L and PD-L1, for example, enhances the ability of DCregs to induce apoptosis and anergy in T cells. These cells also show an elevated secretion of immunoregulatory cytokines such as IL-10 and TGF-β, which are known to promote Treg expansion and contribute to the suppression of effector T cell functions. In addition,DCregs produce various molecules like prostaglandin E2 (PGE2), nitric oxide (NO), and IDO (Indoleamine 2,3-dioxygenase), all of which have profound effects on the immune environment. PGE2 and NO contribute to the overall suppressive milieu, while IDO activity leads to metabolic depletion that inhibits effector T cell functions and supports Treg cell maintenance. Moreover, DCregs interact with Tregs to enhance their suppressive function and stability through mechanisms such as the expression of IDO. They also directly inhibit the activation and proliferation of CD8+ T cells and Th1/Th17 cells, pivotal in controlling inflammation and autoimmunity.

DCregs possess unique suppressive mechanisms that allow them to actively regulate immune responses and promote immune tolerance (see **Figure 4**).

#### Induction of Tregs

DCregs could induce the differentiation and expansion of Tregs, particularly Foxp3^+^ Tregs. By presenting antigens in a tolerogenic manner and providing co-stimulatory signals, DCregs promote the development of Tregs that suppress immune responses and maintain self-tolerance (133–138).

#### Immune checkpoint molecules

Similar to conventional DCs, DCregs express immune checkpoint molecules such as PD-L1and CTLA-4. The engagement of these molecules with their respective receptors on T cells inhibits their activation and proliferation, thereby suppressing immune responses.

#### Production of immunomodulatory cytokines

DCregs secrete immunomodulatory cytokines such as IL-10 and TGF-β (139, 140). These cytokines have potent immunosuppressive effects, including the inhibition of pro-inflammatory cytokine production, suppression of effector T cell responses, and promotion of regulatory T cell function.

#### Indoleamine 2,3-dioxygenase expression

DCregs express the enzyme IDO, which catabolizes tryptophan, an essential amino acid required for T cell proliferation. By depleting tryptophan and generating tryptophan metabolites, DCregs induce a state of tryptophan starvation that inhibits T cell activation and promotes the differentiation of regulatory T cells (139, 141, 142).

#### Modulation of co-stimulatory molecules

DCregs exhibit reduced expression of co-stimulatory molecules such as CD80 and CD86, resulting in impaired T cell activation. This modulation of co-stimulatory signals contributes to the induction of T cell anergy or tolerance (143).

#### Antigen presentation in lymphoid organs

DCregs preferentially migrate to lymphoid organs and present antigens to T cells in a tolerogenic manner. This leads to the induction of antigen-specific tolerance and suppression of immune responses (144).

The extensive data significantly enhances our present comprehension of different subsets of DCregs in the regulation of different conditions. Nevertheless, the primary challenge at present is how to translate our knowledge of DCregs in mouse models to manipulation of human immune system and reveal therapeutic potential of DCregs in human diseases. Promisingly, several research have initiated investigations into the characteristics of DCregs in patients with autoimmune and inflammatory diseases. These studies aim to explore the therapeutic potential of DCregs in the treatment of AID, offering an exciting avenue for further research and potential clinical applications.

The initial investigation on tolerogenic dendritic cells in humans was conducted by Ralph Steinman’s laboratory. Their study involved the subcutaneous administration of antigen-loaded immature dendritic cells to study subjects, with a dosage of 2×10^6^ cells per subject. The treatment was well tolerated by the participants, and the findings showed that the therapy could effectively suppress antigen-specific CD8+ T cell responses for a duration of up to 6 months (145, 146). In a more recent clinical trial, 10 individuals with type 1 diabetes participated, and each subject received four intradermal administrations of 1 × 10^7^ autologous dendritic cells at 2-week intervals. The dendritic cells used in the treatment were modified through the transduction of anti-sense oligonucleotides, which aimed to silence the expression of co-stimulatory molecules such as CD40, CD80, and CD86. However, no specific data regarding the efficacy of the silencing process was reported in the study (129).

The researchers had previously established their silencing protocols in a mouse model of type 1 diabetes (147–149). They demonstrated that the dendritic cell treatments, which involved the silencing of co-stimulatory molecules, had resulted in statistically significant, albeit modest, effects in sparing the progression of the disease in the mouse model (150, 151). Similar to the earlier study conducted by *Steinman*, no adverse events associated with the dendritic cell treatments were reported in this subsequent study (152–154). However, there were limited or no detectable immunological signs of tolerance that could be attributed to the dendritic cell treatments. In conclusion, the study highlights that dendritic cells can acquire a tolerogenic phenotype through various mediators, and these play a significant role in shaping interactions between dendritic cells and naive or effector T cells. Tolerogenic dendritic cells utilize both secreted molecules like IL-10 and retinoic acid, as well as inhibitory receptors, to promote the induction of regulatory T cells (155, 156). Additionally, they provide supplementary signals such as integrins to guide the localization of these developing regulatory T cells to the appropriate anatomical sites. A major challenge in the future application of tolerogenic dendritic cells in immunotherapy will be to carefully select or optimize the specific type(s) of tolerogenic dendritic cells to be used, considering the clinical targets and desired outcomes.

### Regulatory innate lymphoid cells

**Figure 5:**
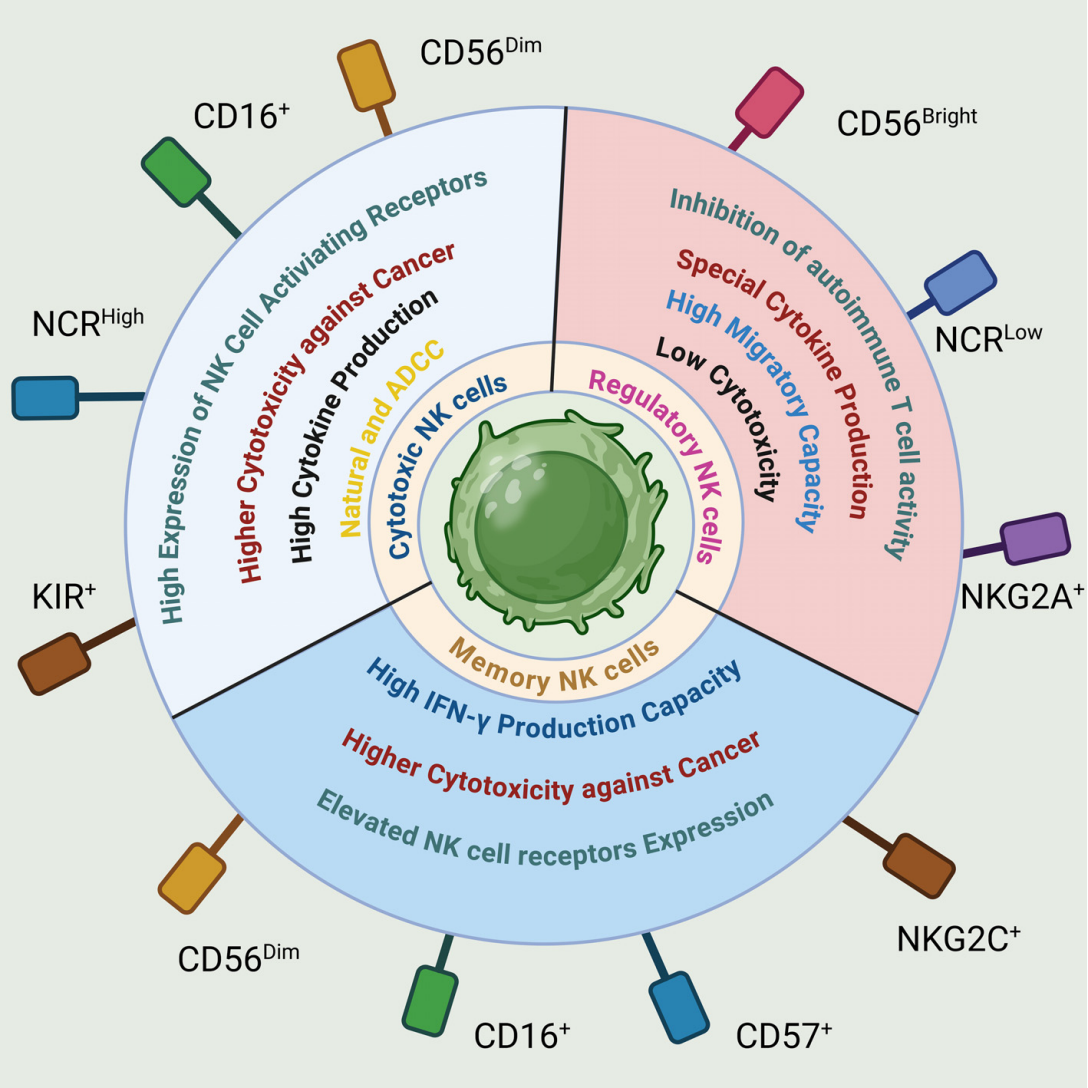
Phenotypical and functional properties of NK cells. NK cells are categorized based on their phenotypic and functional traits, linked to specific receptors like adhesion molecules (CD56, CD57), activating receptors (CD16, NCR, KIR, NKG2C), and inhibitory receptors (NKG2A). These classifications lead to distinct NK cell subsets with regulatory, cytotoxic, or memory functions, each showing unique operational characteristics.

ILCregs are a subset of innate lymphoid cells that possess immunosuppressive functions and play a crucial role in maintaining immune homeostasis. While the concept of ILCregs is still relatively new and evolving, their importance in immune regulation is becoming increasingly recognized (see **Figure 5**).

**Figure 6:**
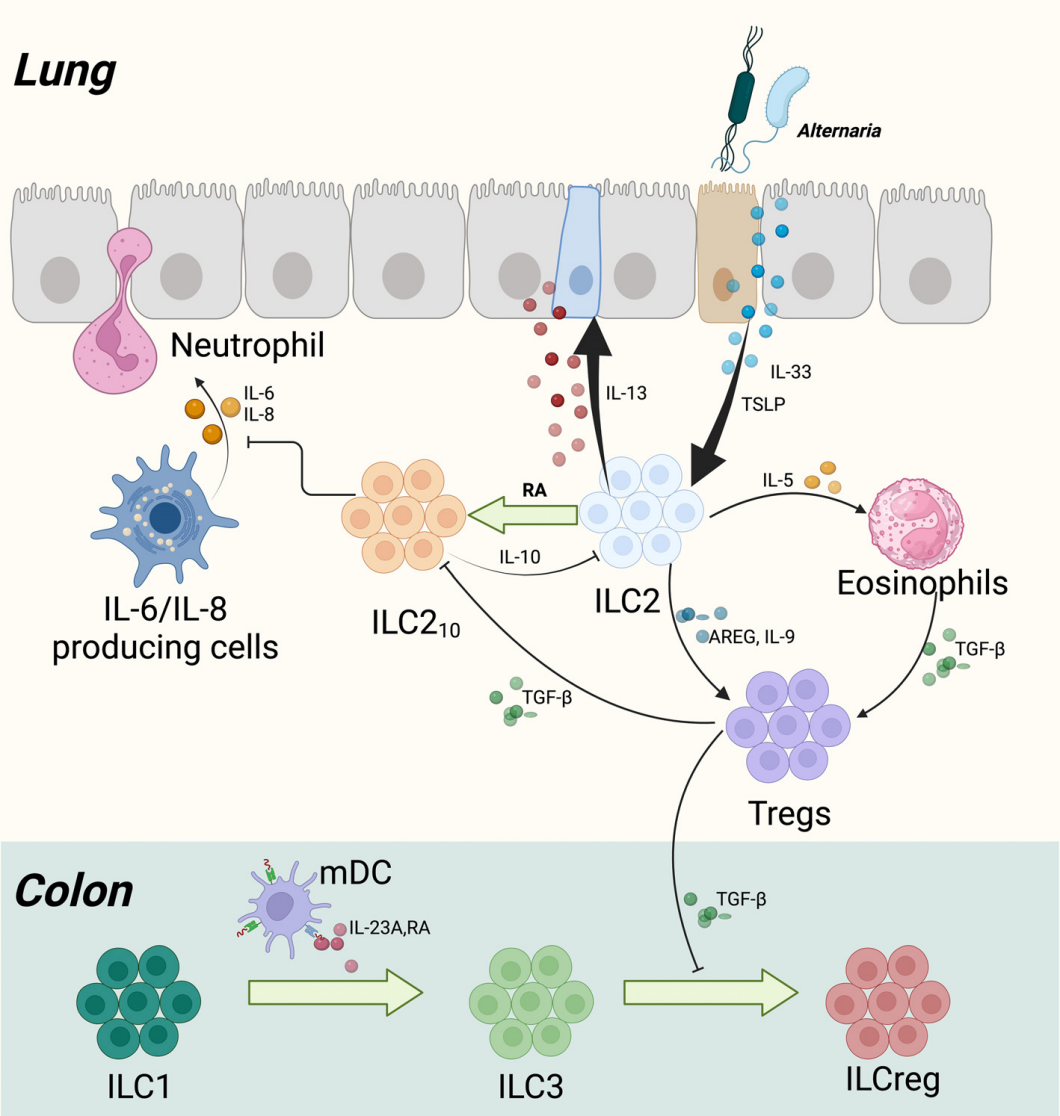
Development of IL-10+ ILCs in the Lung and Colon of Humans. In human lung and colon development, exposure to the fungus Alternaria alternata initiates a sequence of immunological responses starting with activation of the airway epithelium. This activation leads to the release of cytokines such as TSLP (thymic stromal lymphopoietin) and IL-33, which in turn activate type 2 innate lymphoid cells (ILC2s). These cells respond by producing IL-5 and IL-13, contributing to eosinophil recruitment and goblet cell hyperplasia, respectively. Additionally, TSLP is implicated in reducing corticosteroid responsiveness. IL-13 also stimulates the release of retinoic acid (RA) from the epithelium, which facilitates the transformation of ILC2s into a subtype producing IL-10 (ILC2_10_s). These ILC210s play a critical role in dampening type 2 inflammatory responses and enhancing epithelial barrier integrity by reducing IL-6 and IL-8 levels, thereby inhibiting neutrophil migration. Concurrently, Tregs develop and secrete transforming growth factor-beta (TGF-β) to further regulate inflammation and influence the activity of ILC210s. In the colon, CD103+ myeloid dendritic cells (mDCs) release RA and IL-23A, which promote the transformation of CD127+ ILC1s into ILC3s. Tregs support this transition by releasing TGF-β, further promoting the formation of ILCregs.

ILCregs have been identified in various tissues and organs, including kidneys and intestines (21, 157). They are characterized by their ability to produce immuno-suppressive cytokines such as IL-10 and TGF-β, which help dampen excessive immune responses and promote tolerance (21). Similar to other ILC subsets, ILCregs can be classified based on the expression of specific transcription factors and surface markers. Though they lacked expression of the Treg transcription factor Foxp3, ILCregs exert their immune-suppressive effects through multiple mechanisms. In addition to secreting immunosuppressive cytokines, they can interact with other immune cells, such as dendritic cells and T cells to modulate their functions (158). ILCregs can inhibit dendritic cell maturation and antigen presentation, leading to decreased activation of effector T cells. They can also directly interact with T cells, promoting the development of regulatory T cells and suppressing the activity of pro-inflammatory T cell subsets (157) (**Figure 6**).

Unlike the previously described ILCregs, there is also evidence that ILC2s have the capacity to produce IL-10 and may have immunoinhibitory potential (159). Additionally, several pieces of evidence suggest that ILC3s are plastic and can become ILCregs (160). Indeed, the recent findings reveal a regulatory plasticity within all ILC subtypes, and potential crosstalk between DCs and ILCs which should be further investigated in future research.

The study of ILCregs is still in its early stages, and further research is needed to fully understand their ontogeny, functional diversity, and specific roles in different immune contexts. However, their potential as therapeutic targets for immune-mediated diseases and their ability to regulate immune responses make them an exciting area of investigation in immunology.

### Regulatory nature kill cells

While NK cells were initially classified as a uniform population of innate lymphocytes, emerging evidence suggests that NK cells comprise diverse subsets with varying functions, distributions, and developmental origins. Human NK cells in peripheral blood can be categorized into at least two functional subsets based on their expression of CD56 and CD16 (161) (Figure 7). CD56^dim^CD16^+^ NK cells make up approximately 90% of the total NK cells present in the blood. These cells are highly efficient in killing target cells and secrete lower levels of cytokines. On the other hand, regulatory NK cells, namely CD56^bright^ CD16^-^ NK cells, account for less than 10% of total blood NK cells but are enriched in secondary lymphoid tissues (162).

NK cells have been recognized for their ability to carry out effector functions through both direct cytotoxicity and the release of IFN-γ. However, a study conducted by *Perona-Wright* and colleagues has shed light on an additional role of NK cells in attenuating inflammatory processes (163). They demonstrated that NK cells can dampen inflammation by producing IL-10. Therefore, NK cells possess the capacity to not only exert their cytotoxic and pro-inflammatory activities but also contribute to the regulation and resolution of immune responses. Some of the key functions of regulatory NK cells include: (1). Immunomodulation: Regulatory NK cells can interact with other immune cells, such as T cells and dendritic cells, and modulate their activation and function. They can suppress the proliferation and activation of T cells, thereby regulating the adaptive immune response (164–166). (2). Cytokine Production: Regulatory NK cells produce various immunomodulatory cytokines, including IL-10 and TGF-β (167–171). These cytokines have anti-inflammatory properties and can suppress immune responses. (3). Interaction with Dendritic Cells: Regulatory NK cells can interact with dendritic cells and influence their maturation and antigen presentation. This interaction can impact the activation and differentiation of other immune cells, such as T cells (163, 169). (4). Induction of Tolerance: Regulatory NK cells contribute to the induction of immune tolerance, particularly in the context of transplantation and autoimmunity. They can suppress excessive immune responses and promote immune tolerance to self-antigens (172, 173).

Although regulatory NK cells are a relatively less studied subset compared to conventional NK cells, their role in immune regulation is increasingly recognized. Further research is needed to fully understand their precise mechanisms and contribution to immune tolerance and regulation. It’s important to note that regulatory NK cells are still an area of active research, and their clinical applications are being explored in various ongoing studies and clinical trials. As our understanding of their functions and therapeutic potential advances, regulatory NK cells may become an integral part of personalized and targeted immunotherapies in the future.

## Conclusion

In the realm of regulatory immune cells, Tregs, Bregs, MDSCs, DCregs, NKregs, and ILCregs demonstrate their importance in maintaining immune homeostasis and preventing immune-related diseases (13, 49). The interactions and functional overlaps among these cells are manifested in several aspects: First, all these regulatory cells can modulate immune responses by secreting anti-inflammatory and immunosuppressive cytokines like IL-10 and TGF-β. For example, Tregs suppress effector T cells and inflammatory responses by secreting these cytokines, while Bregs similarly use them to suppress excessive humoral responses and promote immune tolerance (13, 28, 49). Moreover, regulatory cells can interact through molecules on their surfaces to jointly suppress immune responses. For instance, Bregs can interact with Tregs or other cells through expressing CD40, CD80, and CD86, thus regulating T cell activation and differentiation (174). DCregs can modulate T cells by engaging PD-1 through expressed PDL-1 (175). Furthermore, regulatory cells can directly or indirectly affect the function of effector cells, such as effector T cells and B cells. Tregs can directly interact with effector T cells to inhibit their activation and proliferation. Similarly, Bregs and DCregs can regulate humoral responses by inhibiting the differentiation and IgG production of effector B cells (128, 176). Additionally, in various disease models, these regulatory cells exhibit similar roles, such as in cancer, autoimmune diseases, and transplantation tolerance. For instance, MDSCs promote tumor immune escape by inhibiting NK and CD8+ T cell activity in the tumor microenvironment, a function similarly exhibited by Tregs (177, 178). Finally, these regulatory cells share signaling pathways in cytokine networks, such as STAT3 and NF-κB, which play key roles in regulating immune responses (179–181). Through these pathways, regulatory cells collaboratively maintain immune homeostasis and prevent overactivation of the immune system. Not only do regulatory immune cells overlap in function, but they also interact through various mechanisms to collectively maintain immune homeostasis and tolerance. A deeper understanding of these cells and their interactions is crucial for developing new immunoregulatory therapeutic strategies.

The expanding research into regulatory immune cells offers a promising frontier for developing novel therapeutic strategies. These diverse roles of immune cells in immune modulation present both opportunities and challenges in translating their functions into effective treatments (182). The clinical applications of regulatory immune cells are vast, ranging from enhancing cancer immunotherapy to preventing autoimmune diseases and improving transplant outcomes (183, 184). As we delve deeper into understanding these cells’ mechanisms and interactions, the potential for innovative treatments grows. These types of cells are pivotal in modulating immune homeostasis, where they prevent autoimmune diseases, mitigate chronic inflammation, and enhance graft tolerance. Therapeutic potential of these cells extends across various domains, including cancer treatment, where manipulation of their numbers and functions can improve outcomes. It underscores the ongoing research into their regulatory mechanisms and their application in treating a wide array of immune-related conditions, necessitating sophisticated strategies to harness their full potential while maintaining the delicate balance of immune responses. However, the clinical application of regulatory immune cells faces significant challenges, including ensuring specificity and selectivity in immune modulation to avoid global immunosuppression, managing long-term safety concerns such as potential chronic infections or oncogenic transformation, and maintaining rigorous standardization and quality control in cell production (49, 185, 186). Ethical and regulatory considerations also play a crucial role, requiring careful management of patient-derived cell manipulations. To overcome these challenges, strategies such as developing targeted therapies that home to specific tissues, leveraging synthetic biology to enhance cell functionality, and optimizing cell preparation and infusion protocols are essential (186, 187). Collaborative efforts across disciplines are required to refine these therapies, ensuring they are safe, effective, and ethically developed. These concerted actions will be pivotal in harnessing the full therapeutic potential of regulatory immune cells, offering innovative treatments for a range of immune-mediated diseases. Future research needs to focus on refining the specificity and safety of therapies that modulate regulatory immune cells, ensuring that they can be integrated effectively into patient care (188–192). This ongoing exploration holds the key to unlocking the full potential of immune regulation in treating a wide array of diseases, potentially revolutionizing our approach to immunotherapy and transplantation medicine.
